# Supplementary material for: Nucleus Near-Infrared (nNIR) Irradiation of Single A549 Cells Induces DNA Damage and Activates EGFR Leading to Mitochondrial Fission
Source: Cells. 2022 Feb 11;11(4):624. doi: 10.3390/cells11040624 (PMC8870661; doi:10.3390/cells11040624)
Supplement: Supplementary file 1 [file cells-11-00624-s001.zip › cells-1561880-supplementary.pdf]

Nucleus near-infrared (nNIR) irradiation of single A549 cells induces DNA damage and activates EGFR leading to mitochondrial fission

Supplementary data

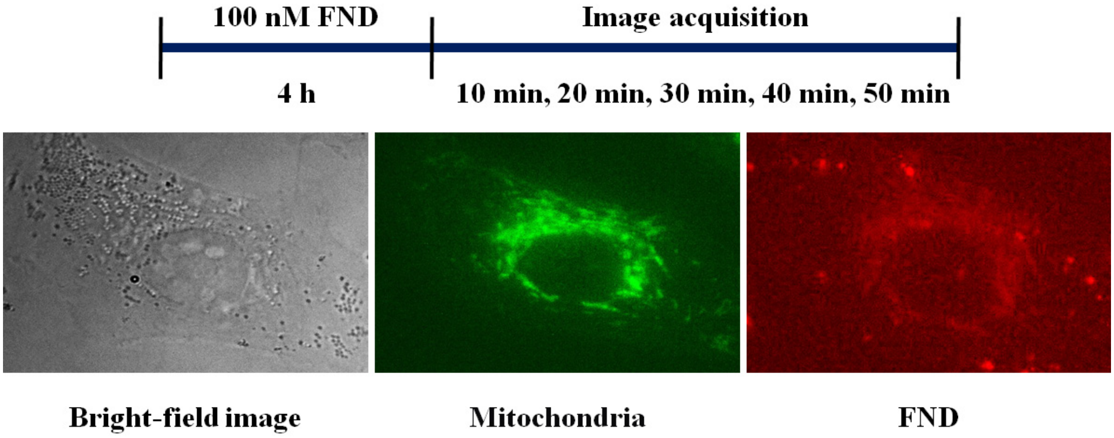

Figure s1. Comparison of the bright-field image of the A549 cell, the fluorescence image of the mitochondrial structure, and the simultaneous FND fluorescence image at 50 min time point. There were scattered FND around the cell membrane but not in the cytosol of the cell.

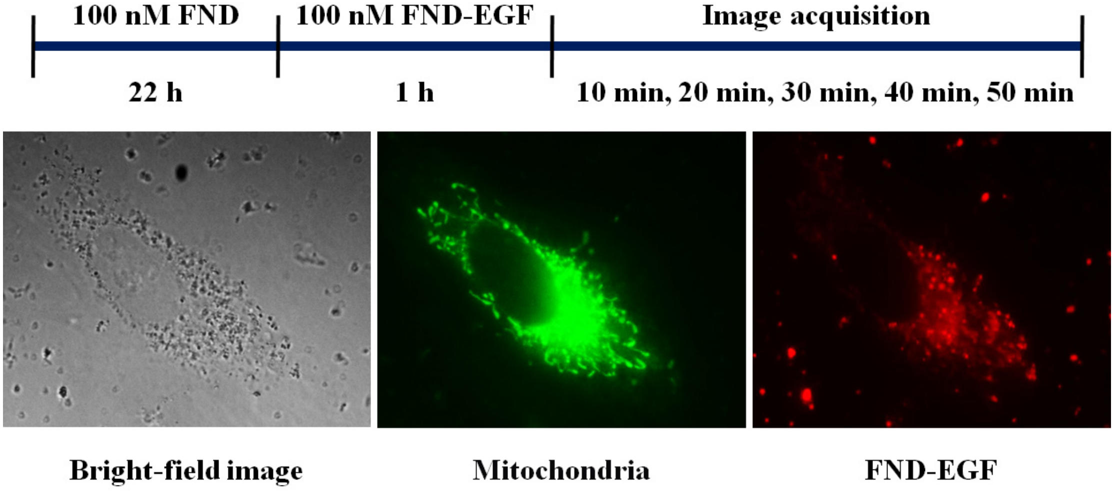

Figure s2. Comparison of the bright-field image of the A549 cell, the fluorescence image of the mitochondrial structure, and the simultaneous FND-EGF fluorescence image at 50 min time point. There was an accumulation of FND-EGF in the cytosol of the cell.
